# Supplementary figures and images for: Human-mediated dispersal of Geniotrigona thoracica (Apidae: Meliponini) colonies promotes high genetic diversity and reduces population structuring in managed populations
Source: PeerJ. 2025 Dec 10;13:e20460. doi: 10.7717/peerj.20460 (PMC12701703; doi:10.7717/peerj.20460)

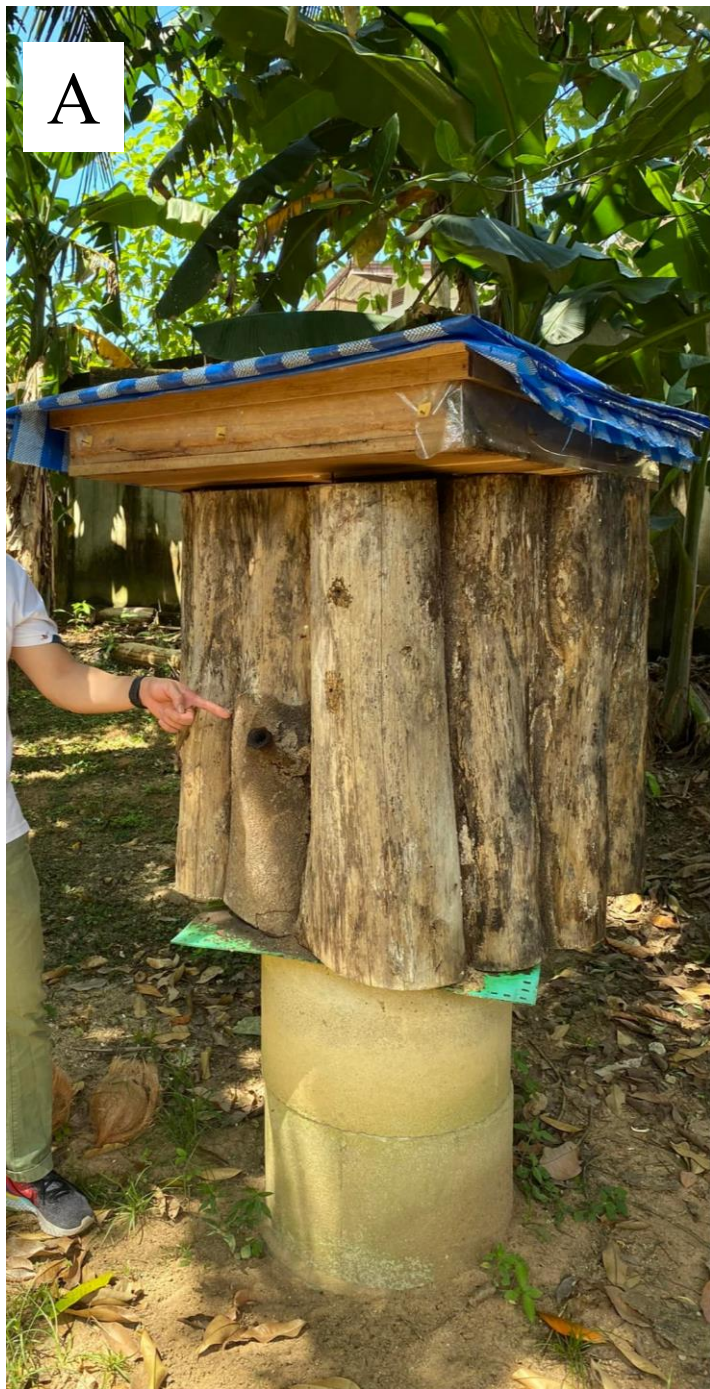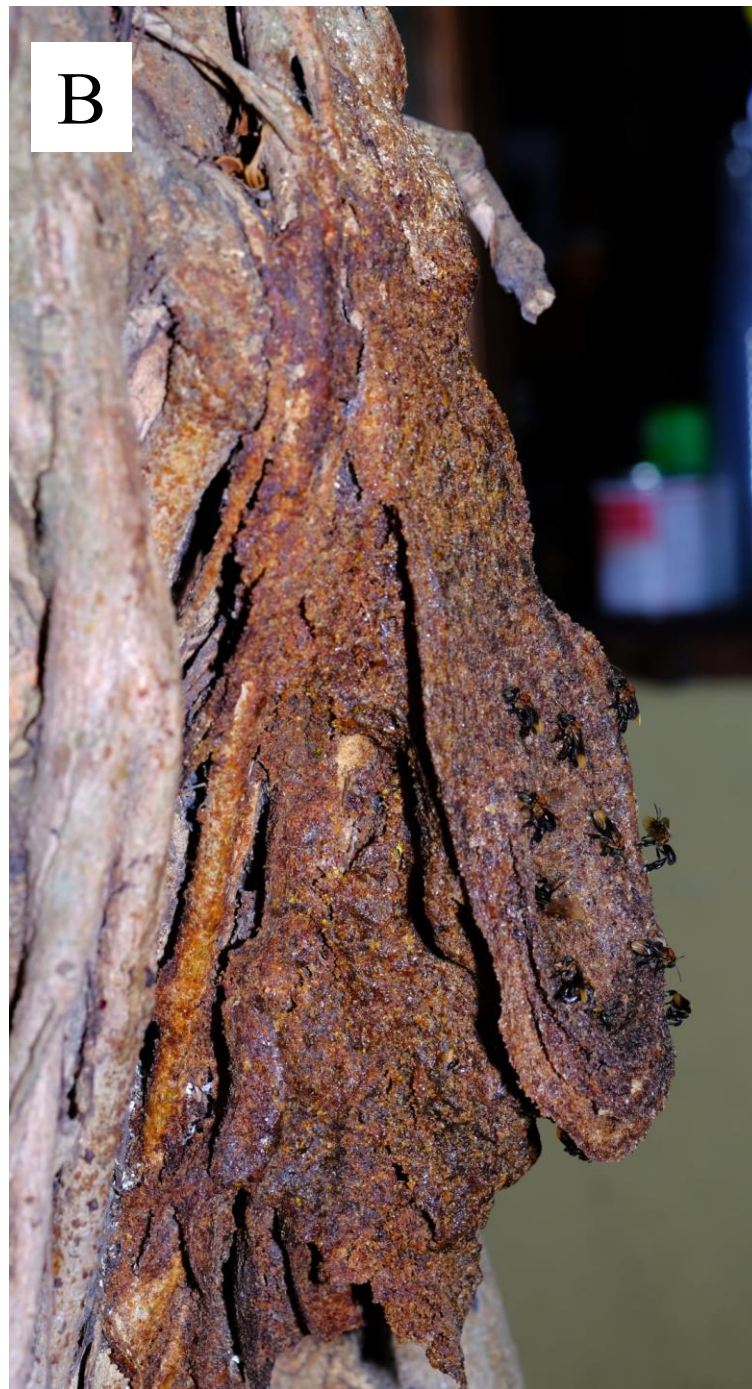

Supplement: Supplemental Information 2 — Colonies of Geniotrigona thoracica maintained in modern commercial hive boxes in Narathiwat Province, southern Thailand (A). The species forms a large, pliable, and resinous entrance tube of propolis at the lower section of the hive box (B). [file peerj-13-20460-s002.pdf]
